# Supplementary material for: Time-resolved certification of frequency-bin entanglement over multi-mode channels
Source: npj Quantum Inf. 2026 Jan 23;12(1):38. doi: 10.1038/s41534-026-01183-5 (PMC12920128; doi:10.1038/s41534-026-01183-5)
Supplement: Supplementary file 1 — Supplementary Information [file 41534_2026_1183_MOESM1_ESM.pdf]

Supplementary Information for: Time-resolved certification of frequency-bin entanglement over multi-mode channels

Stéphane Vinet[1,*], Marco Clementi[2], Marcello Bacchi[2], Yujie Zhang[1], Massimo Giacomin[3], Luke Neal[1], Paolo Villoresi[3], Matteo Galli[2], Daniele Bajoni[4], and Thomas Jennewein[1,5]

[1]Institute for Quantum Computing and Department of Physics & Astronomy, University of Waterloo, 200 University Ave W, Waterloo, Ontario N2L 3G1, Canada
[2]Dipartimento di Fisica "A. Volta", Università di Pavia, Via A. Bassi 6, 27100 Pavia, Italy
[3]Dipartimento di Ingegneria dell'Informazione and Quantum Technologies Research Center, Università degli Studi di Padova, Via Gradenigo 6B, 35131 Padua, Italy
[4]Dipartimento di Ingegneria Industriale e dell'Informazione, Università di Pavia, Via Ferrata 5, 27100 Pavia, Italy
[5]Department of Physics, Simon Fraser University, 8888 University Dr W, Burnaby, British Columbia V5A 1S6, Canada
[*]Corresponding author. Email: svinet@uwaterloo.ca

Supplementary Note 1 - Equatorial measurement

To understand the observed asymmetry between the $\langle\sigma_X\sigma_X\rangle$ and $\langle\sigma_Y\sigma_Y\rangle$ correlations, we simulate in Supplementary Figure 1a the joint temporal intensity (JTI) under the assumption that leakage of the bichromatic pump, enhanced by spontaneous Raman scattering, introduces weak incoherent sidebands at nearby frequencies. These components then interfere to produce an additional modulation at $\Delta\omega/2$ leading to an oscillatory term $\cos\big(\Delta\omega(t_\mathrm{s}+t_\mathrm{i})+\theta_1\big)+\cos\big(\Delta\omega/2(t_\mathrm{s}+t_\mathrm{i})+\theta_2\big)$ along the diagonal of the JTI, consistent with the parity-dependent oscillations observed experimentally in Supplementary Figure 1b. For the bichromatic pump, $J(t_s,t_i)\propto\cos^2(\Delta\omega t_s)\cos^2(\Delta\omega t_i)$ results in a checkered pattern as shown in Supplementary Figure. 1c. This pattern can be seen in the background of the experimental JTI in Supplementary Figure. 1d indicating the presence of uncorrelated pump photons.

Phase tuning

The phase of the equatorial basis measurement can be adjusted either by tuning the joint detection time t_s+t_i or by varying the relative arrival time of the signal and idler photons with respect to the phase modulation signal. The correlation fringes for the latter approach, corresponding to different $\Delta t=t_\mathrm{s}-t_\mathrm{i}$, are plotted in Supplementary Figure. 2. Note that there is a drop in fringe visibility with increasing Δt due to an under-correction of the decay induced by the finite cavity lifetime.

Scalability to higher dimensions

Time-resolved detection provides access to many measurements bases in higher dimensions as discrete frequency-bins produce interference fringes in the joint temporal intensity. For instance, a simulation corresponding to the qutrit projection given in Eq. 1 is presented in Supplementary Figure. 2b.

$$|P_{s,i}(t_s,t_i)\rangle\propto\left(|0_s\rangle+e^{i\Delta\omega t_s}|1_s\rangle+e^{i2\Delta\omega t_s}|2_s\rangle\right)\otimes\left(|0_i\rangle+e^{i\Delta\omega t_i}|1_i\rangle+e^{i2\Delta\omega t_i}|2_i\rangle\right). \quad (1)$$

Supplementary Note 2 - Interferometric measurements

The JTIs for the $\sigma_Z\sigma_Z$ and cross-basis measurements, where one photon is measured in the σ_Z-basis and the other in the equatorial-basis are shown in Supplementary Figure. 3. For the $\sigma_Z\sigma_Z$-basis in Supplementary Figure. 3a, the four JTIs correspond to the $|ij\rangle\langle ij|$ projections, where $i,j\in\{0,1\}$. To be tomographically complete, we must also consider the cross-basis measurements where one photon is measured in the σ_Z- basis and the other in the equatorial basis. We present the JTIs

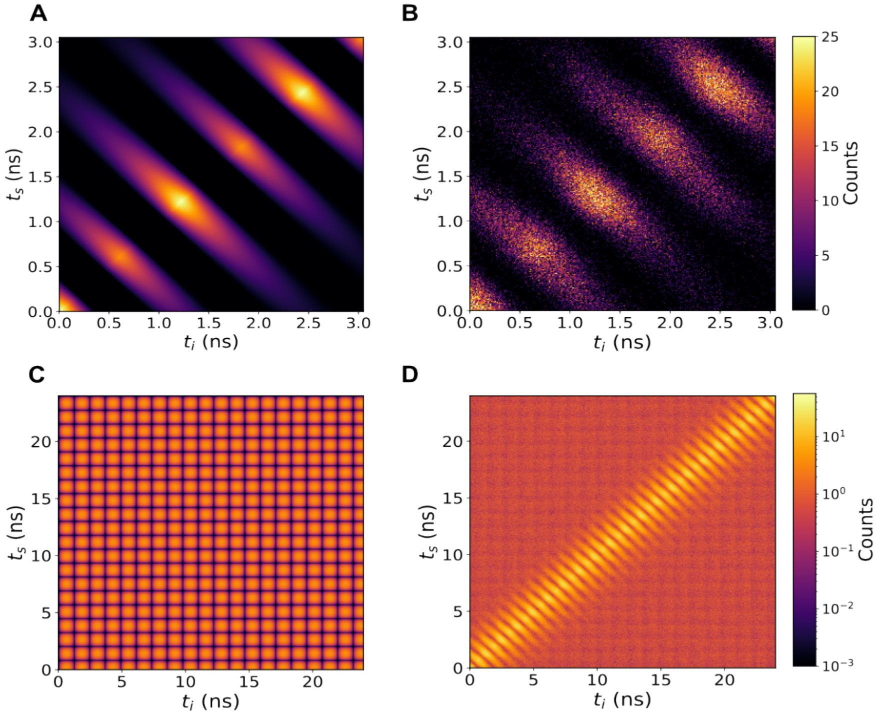

Supplementary Figure 1. Joint temporal intensity. (a) Theoretical JTI for three frequency components each separated by $\Delta\omega/2$. **(b)** Measured JTI over single-mode fiber. **(c)** JTI for a classical bichromatic pump. **(d)** Measured JTI with logarithmic color scale. For panels (c) and (d), the time axes have been rescaled from 3.05 ns to 24 ns.

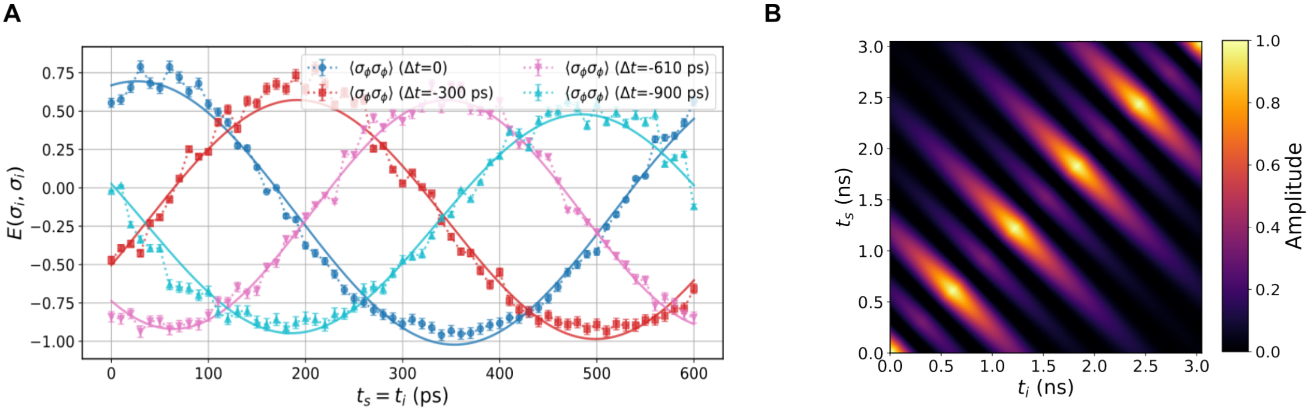

Supplementary Figure 2. $\langle\sigma_\phi\sigma_\phi\rangle$**. (a)** Correlation function $\langle\sigma_\phi\sigma_\phi\rangle$ as a function of the absolute time τ for different relative time delays $\Delta t = t_s - t_i$. Here ϕ denotes the azimuthal angle on the equator of the Poincaré sphere. Due to the cavity lifetime, the amplitude of the oscillation decays proportional to $|t_s - t_i|$ suggesting under-corrections in our time-dependent detector modeling. **(b)** JTI simulation for the time-resolved detection of a qutrit as described in Eq. 1.

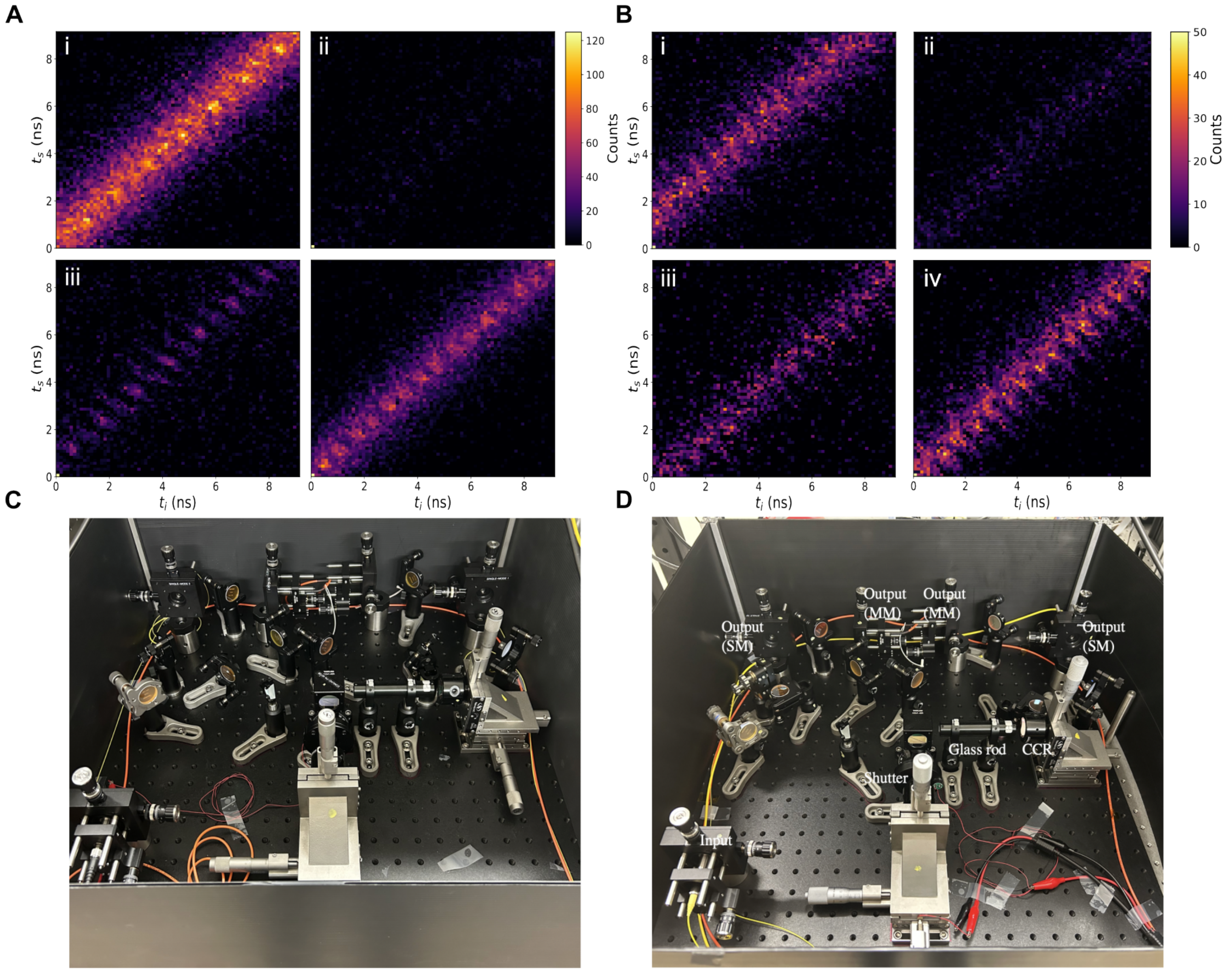

Supplementary Figure 3. σ_Z- **basis measurements** **(a)** $\sigma_Z\sigma_Z$ basis: experimental JTIs in i-iv correspond to the projections $|00\rangle\langle 00|, |01\rangle\langle 01|, |10\rangle\langle 10|, |11\rangle\langle 11|$ respectively. **(b)** i-ii experimental JTIs corresponding to corresponding to equatorial-σ_Z measurements, iii-iv JTIs σ_Z-equatorial basis measurements. Photographs of the **(c)** idler photon's frequency-bin analyzer : the FWI has a single-mode fiber input, **(d)** the signal photon's frequency-bin analyzer: the FWI has a multi-mode fiber input (CCR: corner cube retroreflector).

associated to the signal in the equatorial basis and idler in the σ_Z basis followed by the signal in the σ_Z basis and idler in the equatorial basis in Supplementary Figure. 3b i-iv. By post-selecting on detection times t_i and t_s we can implement projections in the $\sigma_Z\sigma_X, \sigma_Z\sigma_Y, \sigma_X\sigma_Z, \sigma_Y\sigma_Z$ bases as required for quantum state tomography. In our implementation, both interferometers (pictured in Supplementary Figure. 3c-d) operated without active phase stabilization, which constrained the measurement integration time T_{INT}. Indeed, the phase in each interferometer drifts randomly according to environmental fluctuations. If both phase shifts follow a random walk, the variance of the interferometers' relative phase $\Delta\phi(t)$ grows linearly over time which leads to an exponential decay of $\langle\sigma_Z\sigma_Z\rangle$ as observed in Supplementary Figure. 4. Specifically, $\langle\sigma_Z\sigma_Z\rangle(t)$ decays proportionally to $\langle\sigma_Z\sigma_Z\rangle(t=0)\times e^{-2Dt}$, where D is the diffusion constant reflecting the phase noise introduced by the environment.

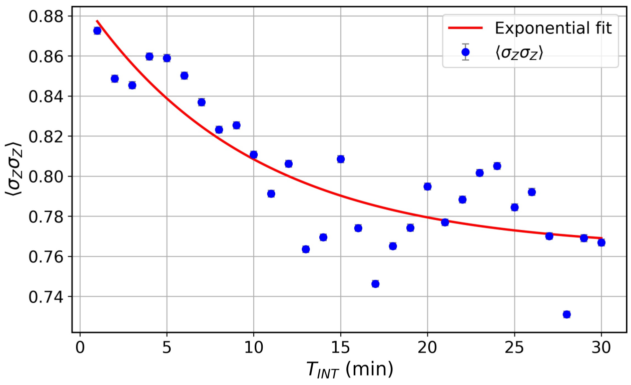

Supplementary Figure 4. $\langle\sigma_Z\sigma_Z\rangle(t)$ **:** phase fluctuations in both interferometers lead to an exponential decay of $\langle\sigma_Z\sigma_Z\rangle$.

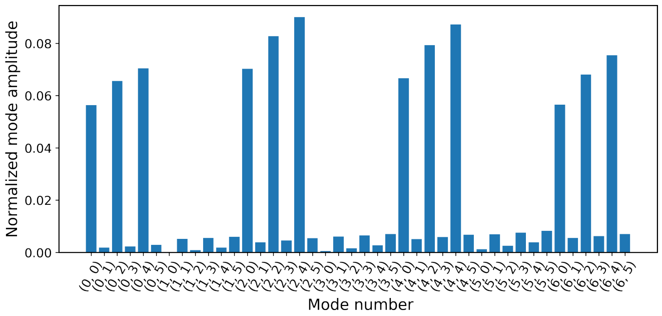

Supplementary Figure 5. Modal decomposition of the point spread function into the first 30 Hermite-Gaussian modes (n,m).

Supplementary Note 3 - Multi-mode characteristics

To characterize the multi-mode channel we image the beam profile in the signal interferometer with a CCD camera (*New Imaging Technologies WiDy SenS 640*) and perform a modal decomposition of the point spread function (PSF). The PSF is expanded into the Hermite-Gaussian (HG) basis and the corresponding mode weights, in Supplementary Figure. 5, provide a measure of how the optical power distribution among the first 30 spatial modes. From these weights, we extract an effective mode number $M \sim 12$ indicating that the beam energy is predominantly shared among the twelve modes with a clear dominance of even-order HG modes consistent with the spatial symmetry of the measured PSF.
